# A two-dimensional Zn coordination polymer with a three-dimensional supra­molecular architecture

**DOI:** 10.1107/S2056989017012452

**Published:** 2017-09-05

**Authors:** Fuhong Liu, Yan Ding, Qiuyu Li, Liping Zhang

**Affiliations:** a Basis Department, Jilin Business and Technology College, Changchun, Jilin, People’s Republic of China

**Keywords:** crystal structure, coordination polymer, zinc complex, two-dimensional layer

## Abstract

The title compound, poly[bis­{μ_2_-4,4′-bis­[(1,2,4-triazol-1-yl)meth­yl]biphenyl-κ^2^ *N* ^4^:*N* ^4′^}bis­(nitrato-κ*O*)zinc(II)], [Zn(NO_3_)_2_(C_18_H_16_N_6_)_2_]_*n*_, is a two-dimensional zinc coordination polymer constructed from 4,4′-bis­[(1*H*-1,2,4-triazol-1-yl)meth­yl]-1,1′-biphenyl units. It was synthesized and characterized by elemental analysis and single-crystal X-ray diffraction. The Zn^II^ cation is located on an inversion centre and is coordinated by two O atoms from two symmetry-related nitrate groups and four N atoms from four symmetry-related 4,4′-bis­[(1*H*-1,2,4-triazol-1-yl)meth­yl]-1,1′-biphenyl ligands, forming a distorted octa­hedral {ZnN_4_O_2_} coordination geometry. The linear 4,4′-bis­[(1*H*-1,2,4-triazol-1-yl)meth­yl]-1,1′-biphenyl ligand links two Zn^II^ cations, generating two-dimensional layers parallel to the crystallographic (132) plane. The parallel layers are connected by C—H⋯O, C—H⋯N, C—H⋯π and π–π stacking inter­actions, resulting in a three-dimensional supra­molecular architecture.

## Abstract 2

The characteristic structural feature of a new two-dimensional Zn coordination polymer is an infinite polymeric layer parallel to the crystallographic (132) plane.

## Chemical context   

Over the past few decades, the self-assembly of coordination polymers (CPs) or metal–organic frameworks (MOFs) based on metal ions or clusters and organic ligands has attracted much attention, owing to their intriguing mol­ecular topologies and potential applications. Multidentate ligands derived from 1,2,4-triazole that contain an aromatic core have been used for this purpose, examples being 1,4-bis­(1*H*-1,2,4-triazol-1-ylmeth­yl)benzene (Wang *et al.*, 2007 ▸; Ding & Zou, 2010 ▸; Zhu *et al.*, 2010 ▸), 1,3-bis­(1*H*-1,2,4-triazol-1-ylmeth­yl)benzene (Zhang *et al.*, 2012 ▸; Ge *et al.*, 2008 ▸; Zhu *et al.*, 2015 ▸), 1,2-bis­(1*H*-1,2,4-triazol-1-ylmeth­yl)benzene (Yang *et al.*, 2009 ▸; Zhao *et al.*, 2017 ▸; Zhang *et al.*, 2013 ▸), 1,3,5-tris­(1*H*-1,2,4-triazol-1-ylmeth­yl)benzene (Li *et al.*, 2012 ▸; Yin *et al.*, 2009 ▸; Shi *et al.*, 2011 ▸), 4,4′-bis­[(1*H*-1,2,4-triazol-1-yl)meth­yl]-1,1′-biphenyl (Mu *et al.*, 2011 ▸; Ren *et al.*, 2010 ▸; Ni *et al.*, 2010 ▸). Hydro­thermal synthesis has been proved to be an effective method for the construction of these new coordination polymers. In this study, a new two-dimensional CP, *viz.* poly[bis­{μ_2_-4,4′-bis­[(1,2,4-triazol-1-yl)meth­yl]biphenyl-κ^2^ *N* ^4^:*N* ^4′^}bis­(nitrato-κ*O*)zinc], [Zn(NO_3_)_2_(C_18_H_16_N_6_)_2_]_*n*_, was synthesized under hydro­thermal conditions by the reaction of Zn(NO_3_)_2_·6H_2_O and 4,4′-bis­[(1*H*-1,2,4-triazol-1-yl)meth­yl]-1,1′-biphenyl at 313 K for 48 h. We report here its crystal structure and its elemental analysis.

## Structural commentary   

The title complex crystallizes in the triclinic space group *P* 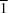; the asymmetric unit of the structure consists of one Zn^II^ cation (site symmetry 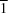), one nitrate anion and one 4,4′-bis­[(1*H*-1,2,4-triazol-1-yl)meth­yl]-1,1′-biphenyl ligand.
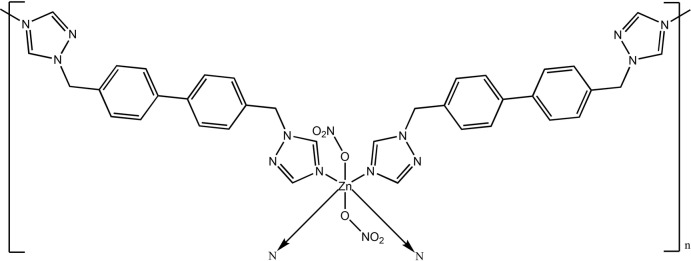


**Figure 1:**
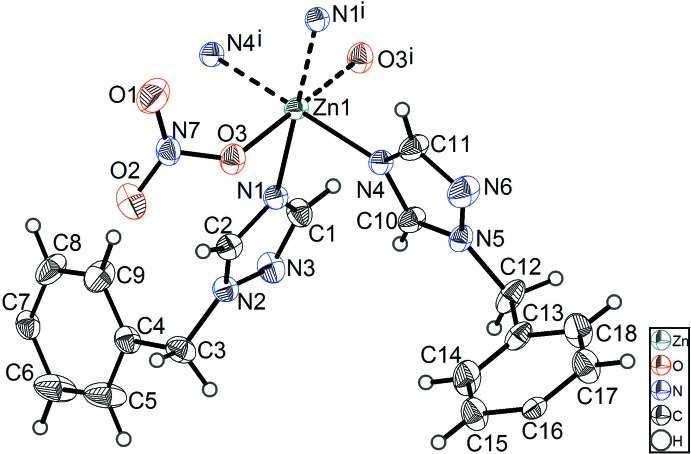
The asymmetric unit of (I), showing the atom-numbering scheme. Displacement ellipsoids drawn at the 25% probability level. [Symmetry code: (i) −*x*, 2 − *y*, −*z*.]

**Figure 2:**
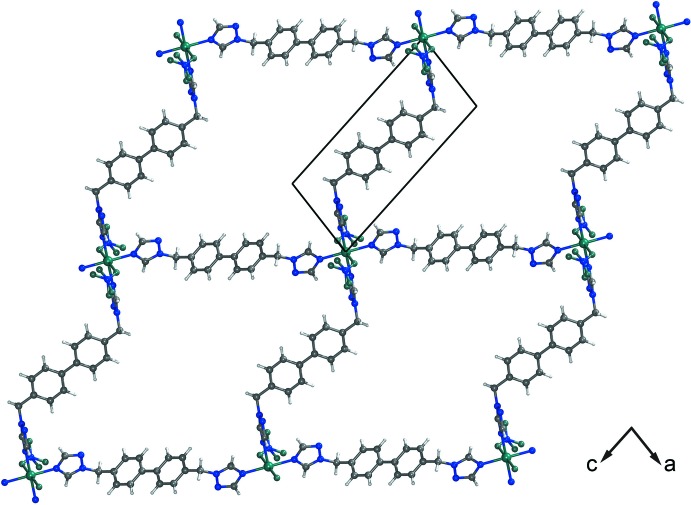
The two-dimensional layer parallel to the crystallographic (132) plane.

As shown in Fig. 1 ▸, each Zn^II^ cation exhibits a slightly distorted octa­hedral {ZnN_4_O_2_} coordination geometry and is coordinated by four N atoms (N1, N4, N1^i^ and N4^i^) from four symmetry-related organic ligands and two O atoms (O3 and O3^i^) from two symmetry-related nitrate groups (see Fig. 1 ▸ for symmetry code). The Zn—O [2.191 (2) Å] and Zn—N bond lengths [2.124 (3)–2.168 (2) Å] are in agreement with corresponding bond lengths found in previously reported Zn^II^ coordination polymers. For the title coordination polymer, the Zn^II^ cation is coordinated by four 4,4′-bis­[(1*H*-1,2,4-triazol-1-yl)meth­yl]-1,1′-biphenyl ligands and two nitrate anions, and each organic ligand in turn connects two Zn^II^ cations to generate a two-dimensional layer parallel to the crystallographic (132) plane. The organic ligand adopts a *cis*,*cis* substituent conformation. The two distinct Zn⋯Zn distances are 18.397 (3) and 18.964 (3) Å (see Fig. 2 ▸). The two benzene rings of the 4,4′-bis­[(1*H*-1,2,4-triazol-1-yl)meth­yl]-1,1′-biphenyl ligand lie nearly in one plane [dihedral angle = 0.00 (2)°]. The two triazole groups of the 4,4′-bis­[(1*H*-1,2,4-triazol-1-yl)meth­yl]-1,1′-biphenyl ligand are inclined to the plane of the central biphenyl groups, with dihedral angles of 80.050 (2) (C1/C2/N1/N2/N3) and 85.511 (2)° (C10/C11/N4/N5/N6). Four adjacent Zn^II^ cations are connected by four linear organic ligands and form a 72-membered macrocyclic ring in the above-mentioned two-dimensional layer (see Fig. 2 ▸).

## Supra­molecular features   

Neighbouring layers are linked to each other by by weak interactions (Table 1 ▸), including C—H⋯O, C—H⋯N, C—H⋯π [C11—H11⋯*Cg*1^ii^ = 3.6756 (8) Å and C12—H12⋯*Cg*2^iii^ = 3.5252 (7) Å; *Cg*1 and *Cg*2 are the centroids of the triazole (C1/C2/N1/N2/N3) and phenyl (C4–C9) rings, respectively; symmetry codes: (ii) 2 − *x*, −*y*, −*z*; (iii) 1 − *x*, 1 − *y*, −*z*] contacts and π–π stacking inter­actions [*Cg*1⋯*Cg*1^ii^ = 3.6296 (10) Å]. These interactions, together with the covalent inter­actions in the infinite two-dimensional polymeric-like layer, make up a three-dimensional supra­molecular structure.

## Database survey   

A search in the Cambridge Structural Database (Groom *et al.*, 2016 ▸) for zinc and the 4,4′-bis­[(1*H*-1,2,4-triazol-1-yl)meth­yl]-1,1′-biphenyl moiety gave eight hits. Seven of them are con­structed by 4,4′-bis­[(1*H*-1,2,4-triazol-1-yl)meth­yl]-1,1′-biphenyl units and different carboxyl­ate ligands. One example is a chain structure based on Zn and 4,4′-bis­[(1*H*-1,2,4-triazol-1-yl)meth­yl]-1,1′-biphenyl (PUQWAA; Ni *et al.*, 2010 ▸).

## Synthesis and crystallization   

Zn(NO_3_)_2_·6H_2_O (0.1 mmol), 4,4′-bis­[(1*H*-1,2,4-triazol-1-yl)meth­yl]-1,1′-biphenyl (0.1 mmol) and water (6 ml) were mixed and placed in a thick Pyrex tube, which was sealed and heated to 413 K for 72 h. After cooling to room temperature, colourless block-shaped crystals (53% yield, based on Zn) suitable for X-ray analysis were obtained. Elemental analysis calculated for C_36_H_32_N_14_O_6_Zn: C 52.59, H 3.92, N 23.85%; found: C 52.23, H 3.74, N 23.49%.

## Refinement   

Crystal data, data collection and structure refinement details are summarized in Table 2 ▸. H atoms bonded to C atoms were positioned geometrically and allowed to ride on their parent atoms, with C—H = 0.93 Å with *U*_iso_(H) = 1.2*U*_eq_(C) for other H atoms. Atoms O1 and O2 of the nitrate group are disordered over two orientations, with occupancies of 0.511 (11) and 0.489 (11), and were refined through the use of SADI, RIGU and SIMU commands.

## Supplementary Material

Crystal structure: contains datablock(s) I. DOI: 10.1107/S2056989017012452/vn2130sup1.cif

Structure factors: contains datablock(s) I. DOI: 10.1107/S2056989017012452/vn2130Isup2.hkl

Supporting information file. DOI: 10.1107/S2056989017012452/vn2130Isup3.mol

CCDC reference: 1564369

Additional supporting information:  crystallographic information; 3D view; checkCIF report

## Figures and Tables

**Table 1:** Hydrogen-bond geometry (Å, °)

*D*—H⋯*A*	*D*—H	H⋯*A*	*D*⋯*A*	*D*—H⋯*A*
C3—H3*B*⋯O1^i^	0.97	2.31	3.2728 (7)	170
C3—H3*B*⋯O1*A* ^i^	0.97	2.33	3.2765 (7)	165
C10—H10⋯O2*A* ^ii^	0.93	2.53	3.0888 (6)	115
C14—H14⋯O2^ii^	0.93	2.46	3.5454 (7)	158
C15—H15⋯N6^iii^	0.93	2.58	3.482 (16)	162

Symmetry codes: (i) 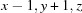; (ii) 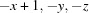; (iii) 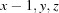.

**Table 2:** Experimental details

Crystal data	
Chemical formula	[Zn(NO_3_)_2_(C_18_H_16_N_6_)_2_]
*M*_r_	822.12
Crystal system, space group	Triclinic, *P* 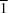
Temperature (K)	293
*a*, *b*, *c* (Å)	7.3257 (15), 9.0188 (18), 15.578 (3)
α, β, γ (°)	81.70 (3), 77.64 (3), 68.90 (3)
*V* (Å^3^)	935.4 (4)
*Z*	1
Radiation type	Mo *K*α
μ (mm^−1^)	0.72
Crystal size (mm)	0.24 × 0.22 × 0.20

Data collection	
Diffractometer	Bruker APEXII Quazar
Absorption correction	Multi-scan (*SADABS*; Krause *et al.*, 2015 ▸)
*T*_min_, *T*_max_	0.84, 0.86
No. of measured, independent and observed [*I* > 2σ(*I*)] reflections	7292, 3271, 2589
*R*_int_	0.042
(sin θ/λ)_max_ (Å^−1^)	0.595

Refinement	
*R*[*F* ^2^ > 2σ(*F* ^2^)], *wR*(*F* ^2^), *S*	0.051, 0.143, 1.05
No. of reflections	3271
No. of parameters	278
No. of restraints	85
H-atom treatment	H-atom parameters constrained
Δρ_max_, Δρ_min_ (e Å^−3^)	0.34, −0.54

Computer programs: *APEX3* (Bruker, 2016 ▸), *SAINT-Plus* (Bruker, 2016 ▸), *SHELXT* (Sheldrick, 2008 ▸), *SHELXTL-Plus* (Sheldrick, 2008 ▸) and *OLEX2* (Dolomanov *et al.*, 2009 ▸).

## supplementary crystallographic information

### Crystal data

**Table:**

[Zn(NO_3_)_2_(C_18_H_16_N_6_)_2_]	*Z* = 1
*M**_r_* = 822.12	*F*(000) = 424
Triclinic, *P*1	*D*_x_ = 1.459 Mg m^−^^3^
*a* = 7.3257 (15) Å	Mo *K*α radiation, λ = 0.71073 Å
*b* = 9.0188 (18) Å	Cell parameters from 7292 reflections
*c* = 15.578 (3) Å	θ = 1.6–25.1°
α = 81.70 (3)°	µ = 0.72 mm^−^^1^
β = 77.64 (3)°	*T* = 293 K
γ = 68.90 (3)°	Block, colorless
*V* = 935.4 (4) Å^3^	0.24 × 0.22 × 0.2 mm

### Data collection

**Table:**

Bruker APEXII Quazar diffractometer	3271 independent reflections
Radiation source: microfocus sealed X-ray tube, Incoatec Iµs	2589 reflections with *I* > 2σ(*I*)
Mirror optics monochromator	*R*_int_ = 0.042
Detector resolution: 7.9 pixels mm^-1^	θ_max_ = 25.0°, θ_min_ = 3.0°
0.5° ω and 0.5° φ scans	*h* = −8→8
Absorption correction: multi-scan (SADABS; Krause *et al.*, 2015)	*k* = −10→9
*T*_min_ = 0.84, *T*_max_ = 0.86	*l* = −18→18
7292 measured reflections	

### Refinement

**Table:**

Refinement on *F*^2^	85 restraints
Least-squares matrix: full	Hydrogen site location: inferred from neighbouring sites
*R*[*F*^2^ > 2σ(*F*^2^)] = 0.051	H-atom parameters constrained
*wR*(*F*^2^) = 0.143	*w* = 1/[σ^2^(*F*_o_^2^) + (0.079*P*)^2^ + 0.2956*P*] where *P* = (*F*_o_^2^ + 2*F*_c_^2^)/3
*S* = 1.05	(Δ/σ)_max_ = 0.001
3271 reflections	Δρ_max_ = 0.34 e Å^−^^3^
278 parameters	Δρ_min_ = −0.54 e Å^−^^3^

### Special details

**Table:**

Geometry. All esds (except the esd in the dihedral angle between two l.s. planes) are estimated using the full covariance matrix. The cell esds are taken into account individually in the estimation of esds in distances, angles and torsion angles; correlations between esds in cell parameters are only used when they are defined by crystal symmetry. An approximate (isotropic) treatment of cell esds is used for estimating esds involving l.s. planes.

### Fractional atomic coordinates and isotropic or equivalent isotropic displacement parameters (Å^2^)

**Table:**

	*x*	*y*	*z*	*U*_iso_*/*U*_eq_	Occ. (<1)
Zn1	0.000000	1.000000	0.000000	0.0406 (2)	
O3	0.1631 (4)	1.1607 (3)	0.00189 (17)	0.0574 (7)	
N1	0.2322 (4)	0.8123 (3)	0.05733 (18)	0.0441 (7)	
N2	0.4790 (5)	0.6937 (4)	0.1274 (2)	0.0482 (7)	
N3	0.4061 (6)	0.5766 (4)	0.1198 (2)	0.0602 (9)	
N4	0.1701 (4)	0.9540 (4)	−0.12813 (18)	0.0457 (7)	
N5	0.3961 (5)	0.8456 (4)	−0.23645 (19)	0.0466 (7)	
N6	0.2787 (6)	0.9865 (5)	−0.2713 (2)	0.0729 (11)	
N7	0.1398 (5)	1.2530 (4)	0.0601 (2)	0.0570 (8)	
C1	0.2590 (6)	0.6550 (5)	0.0771 (3)	0.0554 (10)	
H1	0.179295	0.606514	0.061538	0.066*	
C2	0.3743 (6)	0.8301 (4)	0.0904 (2)	0.0474 (8)	
H2	0.397217	0.925660	0.087958	0.057*	
C3	0.6466 (6)	0.6585 (5)	0.1731 (3)	0.0601 (10)	
H3A	0.687840	0.751186	0.165279	0.072*	
H3B	0.757323	0.571442	0.146296	0.072*	
C4	0.5997 (6)	0.6146 (4)	0.2700 (3)	0.0513 (9)	
C5	0.7455 (7)	0.5120 (7)	0.3132 (4)	0.0902 (17)	
H5	0.873179	0.468413	0.281685	0.108*	
C6	0.7082 (8)	0.4711 (8)	0.4028 (4)	0.0927 (17)	
H6	0.812884	0.404978	0.430318	0.111*	
C7	0.5224 (6)	0.5251 (4)	0.4518 (3)	0.0532 (9)	
C8	0.3756 (8)	0.6273 (6)	0.4079 (3)	0.0813 (16)	
H8	0.246945	0.667746	0.439147	0.098*	
C9	0.4126 (8)	0.6717 (6)	0.3195 (3)	0.0823 (16)	
H9	0.309014	0.741721	0.292602	0.099*	
C10	0.3284 (6)	0.8298 (5)	−0.1520 (2)	0.0515 (9)	
H10	0.384162	0.743198	−0.114162	0.062*	
C11	0.1465 (6)	1.0486 (5)	−0.2035 (2)	0.0588 (10)	
H11	0.046686	1.147437	−0.206804	0.071*	
C12	0.5625 (7)	0.7311 (5)	−0.2909 (3)	0.0658 (13)	
H12A	0.509806	0.678916	−0.324861	0.079*	
H12B	0.641231	0.649898	−0.252615	0.079*	
C13	0.6941 (6)	0.8103 (4)	−0.3527 (3)	0.0530 (10)	
C14	0.8175 (7)	0.8650 (6)	−0.3228 (3)	0.0722 (13)	
H14	0.821652	0.853057	−0.262825	0.087*	
C15	0.9375 (7)	0.9384 (6)	−0.3803 (3)	0.0684 (13)	
H15	1.021917	0.973475	−0.358088	0.082*	
C16	0.9348 (5)	0.9608 (4)	−0.4696 (2)	0.0431 (8)	
C17	0.8101 (7)	0.9040 (6)	−0.4990 (3)	0.0738 (14)	
H17	0.804965	0.915631	−0.558860	0.089*	
C18	0.6915 (8)	0.8296 (6)	−0.4409 (3)	0.0750 (14)	
H18	0.608813	0.792084	−0.462688	0.090*	
O1	−0.0075 (14)	1.3759 (11)	0.0613 (8)	0.0890 (19)	0.500 (12)
O2	0.2754 (14)	1.2075 (12)	0.1048 (6)	0.0792 (17)	0.500 (12)
O1A	−0.0249 (12)	1.3242 (11)	0.1028 (7)	0.0807 (18)	0.500 (12)
O2A	0.2775 (13)	1.2743 (14)	0.0828 (7)	0.0812 (17)	0.500 (12)

### Atomic displacement parameters (Å^2^)

**Table:**

	*U*^11^	*U*^22^	*U*^33^	*U*^12^	*U*^13^	*U*^23^
Zn1	0.0365 (3)	0.0492 (4)	0.0287 (3)	−0.0098 (2)	−0.0025 (2)	0.0033 (2)
O3	0.0658 (17)	0.0635 (16)	0.0492 (15)	−0.0325 (14)	−0.0020 (13)	−0.0084 (13)
N1	0.0444 (16)	0.0499 (17)	0.0331 (15)	−0.0123 (13)	−0.0074 (13)	0.0027 (13)
N2	0.0514 (18)	0.0509 (17)	0.0384 (16)	−0.0136 (14)	−0.0123 (14)	0.0052 (13)
N3	0.077 (2)	0.0481 (18)	0.060 (2)	−0.0216 (17)	−0.0251 (19)	0.0036 (16)
N4	0.0463 (17)	0.0559 (18)	0.0310 (15)	−0.0158 (14)	−0.0048 (13)	0.0013 (13)
N5	0.0506 (17)	0.0545 (17)	0.0347 (16)	−0.0245 (15)	0.0054 (14)	−0.0049 (14)
N6	0.072 (3)	0.088 (3)	0.042 (2)	−0.019 (2)	0.0022 (19)	0.0074 (19)
N7	0.0616 (17)	0.0610 (18)	0.0580 (19)	−0.0299 (14)	−0.0085 (15)	−0.0136 (15)
C1	0.065 (3)	0.052 (2)	0.052 (2)	−0.0185 (19)	−0.022 (2)	−0.0003 (18)
C2	0.052 (2)	0.0469 (19)	0.0385 (19)	−0.0165 (17)	−0.0040 (17)	0.0047 (15)
C3	0.052 (2)	0.071 (3)	0.057 (3)	−0.020 (2)	−0.020 (2)	0.009 (2)
C4	0.059 (2)	0.048 (2)	0.046 (2)	−0.0146 (17)	−0.0201 (18)	0.0056 (17)
C5	0.044 (3)	0.133 (5)	0.070 (3)	−0.017 (3)	−0.018 (2)	0.044 (3)
C6	0.057 (3)	0.133 (5)	0.070 (3)	−0.021 (3)	−0.028 (3)	0.047 (3)
C7	0.071 (3)	0.043 (2)	0.047 (2)	−0.0121 (18)	−0.027 (2)	0.0009 (16)
C8	0.083 (3)	0.077 (3)	0.041 (2)	0.025 (2)	−0.010 (2)	−0.006 (2)
C9	0.091 (4)	0.075 (3)	0.045 (2)	0.021 (3)	−0.027 (2)	0.002 (2)
C10	0.052 (2)	0.055 (2)	0.0356 (19)	−0.0112 (17)	0.0015 (17)	0.0035 (16)
C11	0.049 (2)	0.068 (2)	0.036 (2)	−0.0013 (19)	0.0002 (17)	0.0047 (18)
C12	0.079 (3)	0.055 (2)	0.057 (3)	−0.033 (2)	0.028 (2)	−0.017 (2)
C13	0.058 (2)	0.047 (2)	0.045 (2)	−0.0194 (18)	0.0153 (18)	−0.0088 (17)
C14	0.087 (3)	0.100 (3)	0.034 (2)	−0.048 (3)	0.002 (2)	0.000 (2)
C15	0.075 (3)	0.108 (4)	0.039 (2)	−0.054 (3)	−0.004 (2)	−0.002 (2)
C16	0.0386 (18)	0.0427 (18)	0.0388 (18)	−0.0073 (14)	0.0024 (15)	−0.0055 (15)
C17	0.087 (3)	0.114 (4)	0.038 (2)	−0.060 (3)	−0.007 (2)	0.003 (2)
C18	0.085 (3)	0.111 (4)	0.051 (3)	−0.066 (3)	−0.002 (2)	−0.005 (2)
O1	0.091 (3)	0.077 (3)	0.080 (4)	−0.006 (3)	−0.008 (3)	−0.016 (3)
O2	0.094 (3)	0.080 (4)	0.075 (3)	−0.035 (3)	−0.032 (3)	−0.003 (3)
O1A	0.080 (3)	0.075 (3)	0.074 (4)	−0.020 (3)	0.009 (3)	−0.016 (3)
O2A	0.085 (3)	0.088 (4)	0.087 (4)	−0.041 (3)	−0.030 (3)	−0.008 (3)

### Geometric parameters (Å, º)

**Table:**

Zn1—O3	2.191 (2)	C4—C5	1.367 (6)
Zn1—O3^i^	2.191 (2)	C4—C9	1.376 (6)
Zn1—N1^i^	2.167 (3)	C5—H5	0.9300
Zn1—N1	2.167 (3)	C5—C6	1.385 (7)
Zn1—N4^i^	2.124 (3)	C6—H6	0.9300
Zn1—N4	2.124 (3)	C6—C7	1.363 (7)
O3—N7	1.263 (4)	C7—C7^ii^	1.505 (8)
N1—C1	1.359 (5)	C7—C8	1.376 (6)
N1—C2	1.322 (5)	C8—H8	0.9300
N2—N3	1.372 (4)	C8—C9	1.375 (7)
N2—C2	1.318 (5)	C9—H9	0.9300
N2—C3	1.465 (5)	C10—H10	0.9300
N3—C1	1.314 (5)	C11—H11	0.9300
N4—C10	1.318 (5)	C12—H12A	0.9700
N4—C11	1.353 (5)	C12—H12B	0.9700
N5—N6	1.365 (5)	C12—C13	1.506 (5)
N5—C10	1.310 (5)	C13—C14	1.359 (6)
N5—C12	1.475 (5)	C13—C18	1.364 (6)
N6—C11	1.307 (5)	C14—H14	0.9300
N7—O1	1.237 (8)	C14—C15	1.387 (6)
N7—O2	1.249 (8)	C15—H15	0.9300
N7—O1A	1.237 (7)	C15—C16	1.381 (5)
N7—O2A	1.221 (8)	C16—C16^iii^	1.487 (7)
C1—H1	0.9300	C16—C17	1.375 (5)
C2—H2	0.9300	C17—H17	0.9300
C3—H3A	0.9700	C17—C18	1.390 (6)
C3—H3B	0.9700	C18—H18	0.9300
C3—C4	1.501 (6)		
			
O3—Zn1—O3^i^	180.0	C5—C4—C3	120.3 (4)
N1—Zn1—O3^i^	92.22 (11)	C5—C4—C9	116.5 (4)
N1^i^—Zn1—O3	92.22 (11)	C9—C4—C3	123.2 (4)
N1^i^—Zn1—O3^i^	87.78 (11)	C4—C5—H5	119.1
N1—Zn1—O3	87.78 (11)	C4—C5—C6	121.8 (5)
N1^i^—Zn1—N1	180.0	C6—C5—H5	119.1
N4—Zn1—O3^i^	94.65 (10)	C5—C6—H6	119.1
N4^i^—Zn1—O3	94.65 (10)	C7—C6—C5	121.9 (5)
N4—Zn1—O3	85.35 (10)	C7—C6—H6	119.1
N4^i^—Zn1—O3^i^	85.35 (10)	C6—C7—C7^ii^	122.4 (5)
N4^i^—Zn1—N1^i^	90.36 (12)	C6—C7—C8	116.2 (4)
N4^i^—Zn1—N1	89.64 (12)	C8—C7—C7^ii^	121.4 (5)
N4—Zn1—N1	90.36 (12)	C7—C8—H8	118.9
N4—Zn1—N1^i^	89.64 (12)	C9—C8—C7	122.2 (4)
N4^i^—Zn1—N4	180.0	C9—C8—H8	118.9
N7—O3—Zn1	128.5 (2)	C4—C9—H9	119.3
C1—N1—Zn1	130.5 (3)	C8—C9—C4	121.4 (4)
C2—N1—Zn1	126.5 (2)	C8—C9—H9	119.3
C2—N1—C1	102.7 (3)	N4—C10—H10	124.8
N3—N2—C3	120.7 (3)	N5—C10—N4	110.5 (4)
C2—N2—N3	109.9 (3)	N5—C10—H10	124.8
C2—N2—C3	129.4 (3)	N4—C11—H11	123.6
C1—N3—N2	102.0 (3)	N6—C11—N4	112.9 (4)
C10—N4—Zn1	128.1 (3)	N6—C11—H11	123.6
C10—N4—C11	103.9 (3)	N5—C12—H12A	109.2
C11—N4—Zn1	128.0 (3)	N5—C12—H12B	109.2
N6—N5—C12	122.6 (3)	N5—C12—C13	112.2 (3)
C10—N5—N6	109.0 (3)	H12A—C12—H12B	107.9
C10—N5—C12	128.3 (4)	C13—C12—H12A	109.2
C11—N6—N5	103.8 (3)	C13—C12—H12B	109.2
O1—N7—O3	115.4 (6)	C14—C13—C12	121.5 (4)
O1—N7—O2	130.6 (7)	C14—C13—C18	118.1 (4)
O2—N7—O3	114.0 (5)	C18—C13—C12	120.4 (4)
O1A—N7—O3	122.8 (5)	C13—C14—H14	119.5
O2A—N7—O3	123.5 (6)	C13—C14—C15	121.0 (4)
O2A—N7—O1A	113.6 (7)	C15—C14—H14	119.5
N1—C1—H1	122.6	C14—C15—H15	119.2
N3—C1—N1	114.8 (3)	C16—C15—C14	121.7 (4)
N3—C1—H1	122.6	C16—C15—H15	119.2
N1—C2—H2	124.7	C15—C16—C16^iii^	120.9 (4)
N2—C2—N1	110.7 (3)	C17—C16—C15	116.7 (3)
N2—C2—H2	124.7	C17—C16—C16^iii^	122.4 (4)
N2—C3—H3A	108.9	C16—C17—H17	119.4
N2—C3—H3B	108.9	C16—C17—C18	121.2 (4)
N2—C3—C4	113.5 (3)	C18—C17—H17	119.4
H3A—C3—H3B	107.7	C13—C18—C17	121.4 (4)
C4—C3—H3A	108.9	C13—C18—H18	119.3
C4—C3—H3B	108.9	C17—C18—H18	119.3

Symmetry codes: (i) −*x*, −*y*+2, −*z*; (ii) −*x*+1, −*y*+1, −*z*+1; (iii) −*x*+2, −*y*+2, −*z*−1.

### Hydrogen-bond geometry (Å, º)

**Table:**

*D*—H···*A*	*D*—H	H···*A*	*D*···*A*	*D*—H···*A*
C3—H3*B*···O1^iv^	0.97	2.31	3.2728 (7)	170
C3—H3*B*···O1*A*^iv^	0.97	2.33	3.2765 (7)	165
C10—H10···O2*A*^v^	0.93	2.53	3.0888 (6)	115
C14—H14···O2^v^	0.93	2.46	3.5454 (7)	158
C15—H15···N6^vi^	0.93	2.58	3.482 (16)	162

Symmetry codes: (iv) *x*−1, *y*+1, *z*; (v) −*x*+1, −*y*, −*z*; (vi) *x*−1, *y*, *z*.
